# *trans*-Acting Arginine Residues in the AAA+ Chaperone ClpB Allosterically Regulate the Activity through Inter- and Intradomain Communication*

**DOI:** 10.1074/jbc.M114.608828

**Published:** 2014-09-24

**Authors:** Cathleen Zeymer, Sebastian Fischer, Jochen Reinstein

**Affiliations:** From the Department of Biomolecular Mechanisms, Max Planck Institute for Medical Research, 69120 Heidelberg, Germany

**Keywords:** Allosteric Regulation, Arginine Finger, ATPase, ATPases Associated with Diverse Cellular Activities (AAA), Molecular Chaperone, ClpB, Hsp104, Nucleotide Binding, Oligomerization

## Abstract

The molecular chaperone ClpB/Hsp104, a member of the AAA+ superfamily (ATPases associated with various cellular activities), rescues proteins from the aggregated state in collaboration with the DnaK/Hsp70 chaperone system. ClpB/Hsp104 forms a hexameric, ring-shaped complex that functions as a tightly regulated, ATP-powered molecular disaggregation machine. Highly conserved and essential arginine residues, often called arginine fingers, are located at the subunit interfaces of the complex, which also harbor the catalytic sites. Several AAA+ proteins, including ClpB/Hsp104, possess a pair of such *trans*-acting arginines in the N-terminal nucleotide binding domain (NBD1), both of which were shown to be crucial for oligomerization and ATPase activity. Here, we present a mechanistic study elucidating the role of this conserved arginine pair. First, we found that the arginines couple nucleotide binding to oligomerization of NBD1, which is essential for the activity. Next, we designed a set of covalently linked, dimeric ClpB NBD1 variants, carrying single subunits deficient in either ATP binding or hydrolysis, to study allosteric regulation and intersubunit communication. Using this well defined environment of site-specifically modified, cross-linked AAA+ domains, we found that the conserved arginine pair mediates the cooperativity of ATP binding and hydrolysis in an allosteric fashion.

## Teaser

**Background:** Two neighboring, *trans*-acting arginines in the N-terminal AAA+ domain are essential for oligomerization and activity of ClpB/Hsp104.

**Results:** Both arginines couple nucleotide binding to oligomerization and allosterically regulate the ATPase activity.

**Conclusion:** Site-specifically engineered, cross-linked dimers of AAA+ subunits can be utilized to study allosteric regulation.

**Significance:** This study elucidates the mechanistic role of an essential arginine pair conserved in different AAA+ proteins.

## Introduction

The molecular disaggregation machine ClpB/Hsp104 (caseinolytic peptidase B/heat shock protein 104) is crucial for maintaining protein homeostasis because it reactivates aggregated proteins under cellular stress conditions in concert with the DnaK/Hsp70 chaperone system (1–4). Belonging to the superfamily of AAA+ proteins (ATPases associated with various cellular activities), ClpB/Hsp104 functions as a hexameric complex that converts the chemical energy from ATP hydrolysis into mechanical force (5). Protein disaggregation by ClpB/Hsp104 involves the threading of single polypeptide chains out of the aggregate through the central pore of the hexameric ring (6). High-resolution structural information is available for ClpB from *Thermus thermophilus*, showing a domain architecture that consists of a small N-terminal domain and two highly conserved AAA+ domains, also called nucleotide binding domains (NBD1^2^ and NBD2), per monomer (7). There is a long helical insertion into NBD1, named the M-domain, which was recently identified as a major regulatory element as well as the interaction site for the co-chaperone DnaK/Hsp70 (8–13).

The ATPase modules NBD1 and NBD2 are the motors that drive the molecular machine in a cooperative fashion. The catalytic sites, which are located at the interface between two subunits in the hexameric complex, are built up by highly conserved motifs, namely the Walker A and B motifs that are crucial for nucleotide binding and ATP hydrolysis, respectively (14, 15). Furthermore, there are essential arginine residues, often termed arginine fingers, that contribute to the active sites in *trans* because they are in close proximity to the nucleotide bound to the adjacent subunit. The role of such conserved arginines in AAA+ proteins has been investigated extensively (16–19). However, it is not trivial to distinguish a truly catalytic arginine finger, as initially identified in GTPase-activating proteins (20), from conserved arginines that either stabilize the hexameric state or are crucial for allosteric regulation. The complexity is even increased by the fact that several AAA+ proteins, such as ClpB/Hsp104, ClpA, ClpC, and p97/VCP/Cdc48, possess two highly conserved, neighboring arginines in their NBD1 subunit interface. With this study, we aimed at understanding the mechanistic role of this essential, *trans*-acting pair of arginines in allosteric communication between AAA+ subunits.

We showed previously that NBD1-M and NBD2 of ClpB from *T. thermophilus* can be expressed and purified separately (21), which allowed a detailed and quantitative characterization of both AAA+ motor domains with regard to nucleotide binding, oligomerization, and activity (22–25). Here, we used the construct NBD1-M. Inspired by successful work on the mechanism of ClpX, another AAA+ protein (26–28), we applied a combined approach of covalently linking NBD1-M subunits and introducing Walker A/B and arginine finger mutations. Using these fixed and well determined arrangements of wild-type and mutated subunits in a direct neighborhood, it was possible to dissect the mechanisms of allosteric regulation and intersubunit communication in the AAA+ chaperone ClpB/Hsp104.

## EXPERIMENTAL PROCEDURES

### 

#### 

##### Mutagenesis, Protein Expression, and Purification

The construct ClpB NBD1-M containing amino acids 141–534 of ClpB from *T. thermophilus* as well as the full-length protein ClpB from *T. thermophilus* were described previously (25, 29). Site-directed mutagenesis was performed using QuikChange PCR according to the QuikChange protocol (Agilent Technologies, Santa Clara, CA) and verified by DNA sequencing (Eurofins MWG Operon, Ebersberg, Germany). The mutations P221C, M394C, Q184C, A390C, R322A, R323A, K204Q, and E271Q were introduced as single mutations and/or in various pairwise combinations. Full-length ClpB and the truncated variant NBD1-M were expressed recombinantly in *Escherichia coli* BL21 (DE3) RIL and purified as described previously (25, 29). 5 mm β-mercaptoethanol was added to all buffers for the purification of cysteine-containing variants. Purified proteins were stored in buffer A (50 mm Tris/HCl, pH 7.5, 50 mm KCl, 5 mm MgCl_2_, and 2 mm EDTA), including 5 mm β-mercaptoethanol for cysteine-containing variants.

##### Intermolecular Disulfide Bond Formation to Generate Covalently Linked ClpB Dimers

Cysteine residues were introduced to facilitate the formation of intermolecular disulfide bonds in the ClpB NBD1 subunit interface. The design was based on an available planar hexameric model of ClpB (30). Prior to the reaction, the reducing agent β-mercaptoethanol was removed by buffer exchange. The formation of covalently linked dimers of full-length ClpB or NBD1-M variants using the single cysteine mutant pair P221C/M394C was performed in 50 mm Tris/HCl, pH 7.5, 50 mm KCl, 5 mm MgCl_2_. The buffer was EDTA-free because 50 μm copper phenanthroline was used as the oxidizing agent. Equimolar amounts of the respective cysteine variants (50 μm each) were used in a 5-ml reaction volume. 2 mm ADP was added to trigger oligomerization of ClpB variants. The mixture was incubated for 1 h at 37 °C. Subsequently, the reaction mixture was applied to a Superdex 200 26/60 size exclusion column equilibrated with buffer A to separate the formed dimer from unreacted monomer. The purity of the dimer products was evaluated by non-reducing SDS-PAGE. Test reactions were performed to ensure that homodimer formation was negligible. Another pair of cysteines (Q184C/A390C) was also used to form a covalently linked dimer as described above. However, because this cross-linked variant showed severely impaired ATPase activity, it was not considered for further experiments.

##### Steady-state ATPase Measurements

Steady-state ATPase activity was measured in a coupled colorimetric assay at 25 °C using a JASCO V-650 spectrophotometer (JASCO Germany GmbH, Gross-Umstatt, Germany). ClpB NBD1-M variants were incubated at 25 °C in assay buffer (50 mm Tris/HCl, pH 7.5, 100 mm KCl, 2 mm EDTA, 0.4 mm phosphoenolpyruvate, 0.4 mm NADH, 0.1 g/liter BSA, 4 units/ml pyruvate kinase, 6 units/ml lactate dehydrogenase, and 10 mm MgCl_2_). Importantly, reducing agents were strictly excluded from the assay buffer to maintain the intermolecular disulfide bonds of covalently linked dimers. The reaction was started by adding ATP (0.01–8 mm). Measurements were performed for protein concentrations of 1–30 μm (with respect to monomeric units). The decreasing absorption at 340 nm was monitored over time, and the maximal slope was used to determine the ATPase turnover rate per monomer (ϵ(NADH) = 6220 m^−1^ cm^−1^). The data were analyzed with the Hill equation (Equation 1) using the program GraphPad Prism version 5.0.

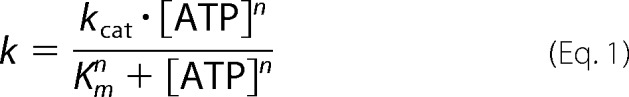


##### Stopped Flow Experiments (Determination of MANT-dADP Binding Parameters)

Nucleotide binding experiments were performed with a BioLogic SFM-400 stopped flow instrument in single mixing configuration (BioLogic Science Instruments, Claix, France) in buffer A at 25 °C essentially as described previously (25). The fluorescently labeled nucleotide MANT-dADP was purchased from BIOLOG (Bremen, Germany). The excitation wavelength was set to 296 nm, and the fluorescence signal was observed using a 400-nm long pass filter (400FG03-25, LOT Oriel Group). This setup was used to selectively excite protein-bound MANT-dADP via fluorescence resonance energy transfer (FRET) from the initially excited tryptophan residues of the protein. Kinetic traces were recorded as triplicates and averaged. Data analysis was performed using the program GraphPad Prism version 5.0.

Kinetic traces from direct binding experiments (2 μm ClpB NBD1-M mixed 1:1 with 10–50 μm MANT-dADP) were fitted to exponential functions. The extracted rate constants were plotted against the nucleotide concentration. The association rate constants *k*_on_ for MANT-dADP binding were obtained from the slope of the resulting linear functions. Kinetic traces from dissociation experiments (2 μm ClpB NBD1-M incubated with 15 μm MANT-dADP and subsequently mixed 1:1 with 5 mm Mg-ADP) were fitted to exponential functions; the extracted rate constants correspond to the dissociation rate constants *k*_off_ of MANT-dADP binding. The *K_D_* was calculated from the ratio *k*_off_/*k*_on_. Given protein concentrations refer to monomeric units.

##### Fluorescence Equilibrium Titrations (Determination of ADP/ATP Binding Parameters)

Fluorescence titrations were performed at 25 °C in buffer A using a JASCO FP-8500 fluorescence spectrometer (JASCO Germany GmbH) as described previously (25). The excitation wavelength was set to 296 nm to facilitate selective excitation of protein-bound MANT-dADP via FRET from nearby tryptophan residues. The MANT fluorescence signal was monitored at 441 nm. Direct titrations of ClpB NBD1-M variants (at 2 or 20 μm) with MANT-dADP (2–50 μm) were used to determine the binding affinity of MANT-dADP, which was subsequently applied as the reference *K_D_* in displacement titrations to determine *K_D_*(ADP) or *K_D_*(ATP). Here, ClpB NBD1-M variants (at 2 or 20 μm) were incubated with MANT-dADP (15–40 μm) and subsequently titrated with ADP (2.5–300 μm) or ATP (125–20,000 μm). ATP titrations were performed in the presence of 2 mm phosphoenolpyruvate and 0.01 mg/ml pyruvate kinase (Roche Applied Science) as an ATP-regenerating system. The data were corrected for dilution effects and analyzed with a cubic equation for competing ligands using the initial concentrations of protein and MANT-dADP as well as the *K_D_*(MANT-dADP) from the direct titration as input values (31). The program GraFit version 5.0 was used for data fitting.

##### Gel Filtration Experiments with Static Light Scattering (SLS) Analysis

Gel filtration experiments were performed on a Superdex 200 10/300 GL column connected to a refractive index detector (2414 from Waters (Milford, MA)), a photodiode array detector (2996 from Waters), and a multiangle light scattering detector (Dawn Heleos, Wyatt (Santa Barbara, CA)) in buffer A as described previously (25). The running buffer was either nucleotide-free or supplemented with 2 mm ADP or 2 mm ATP. 40 μl of 100 μm NBD1-M (with respect to monomeric units) were injected, resulting in a final concentration of about 2 μm at the detector due to a 1:50 dilution by the gel filtration column. Molecular mass values were extracted from the multiangle light scattering data using the ASTRA software (Wyatt).

##### Dynamic Light Scattering (DLS) Experiments

DLS experiments were performed at protein concentrations of 1–30 μm (with respect to monomeric units) in buffer A, which was either nucleotide-free or supplemented with 2 mm ADP or 2 mm ATP, respectively. In the case of ATP, 2 mm phosphoenolpyruvate and 0.01 mg/ml pyruvate kinase (Roche Applied Science) were present as an ATP-regenerating system. The measurements were performed with a Viscotek 802DAT DLS instrument (Viscotek, Waghäusel, Germany). 40 scans with a measuring time of 5 s/scan were recorded. Hydrodynamic radii and molecular mass values were extracted from the DLS data using the OmniSIZE version 3.0 software package.

##### Disaggregation Assay (Chaperone-assisted Reactivation of Heat-aggregated α-Glucosidase)

The assay was performed to test whether disulfide cross-linking using the cysteine pair P221C/M394C affects the chaperone activity of full-length ClpB. 0.2 μm α-glucosidase from *Bacillus stearothermophilus* was denatured for 8 min at 75 °C in reaction buffer containing 50 mm MOPS, pH 7.5, 150 mm KCl, 10 mm MgCl_2_, and 5 mm ATP. Chaperones were added prior to refolding at 55 °C. The total chaperone concentrations were *c*(ClpB) = 1.0 μm, *c*(DnaK) = 1.6 μm, *c*(DnaJ) = 0.5 μm, and *c*(GrpE) = 0.2 μm. The co-chaperones DnaK, DnaJ, and GrpE from *T. thermophilus* were expressed and purified as described previously (32). Samples were taken after 30, 60, and 120 min and diluted 1:10 into assay buffer containing 50 mm KP_i_, pH 6.8, 2 mm *para*-nitrophenyl-α-d-glucopyranoside, 0.1 mg/ml BSA. The α-glucosidase activity was measured at 40 °C using a microplate spectrophotometer (Varioskan, Thermo Electron, Vantaa, Finland). The average rate of absorption increase at 405 nm was monitored and normalized against a positive control containing α-glucosidase that was not heat-aggregated. Importantly, reducing agents were strictly excluded from all buffers to maintain the intermolecular disulfide bonds of covalently linked ClpB dimers.

## RESULTS

### 

#### 

##### Conserved Arginines in ClpB NBD1 Mediate the Coupling between Nucleotide Binding and Oligomerization

**FIGURE 1.**
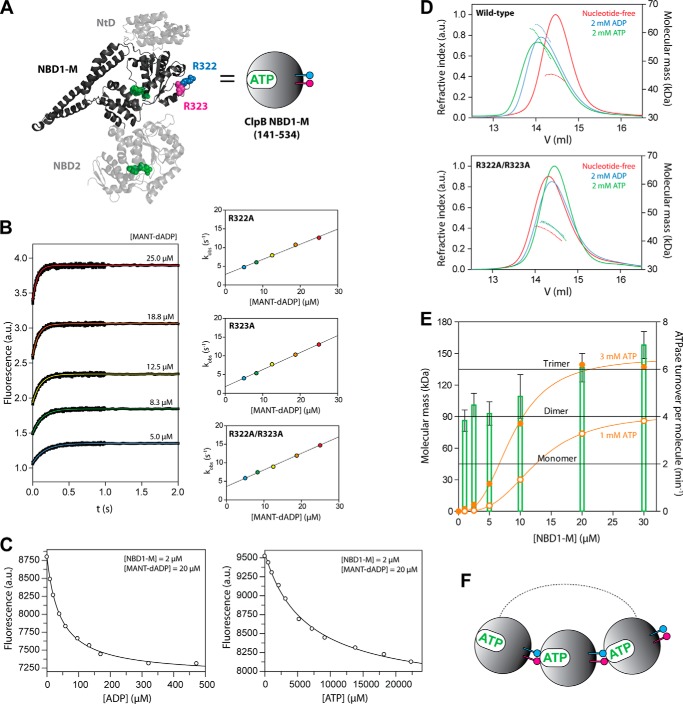
**Characterization of ClpB NBD1-M wild type, single mutants R322A and R323A, and double mutant R322A/R323A.** *A*, the ClpB construct NBD1-M comprises amino acids 141–534, which are highlighted in *dark gray* in the crystal structure of full-length ClpB (Protein Data Bank code 1QVR). The schematic representation for this construct is a *gray sphere*. ATP is shown in *green*. The conserved arginines Arg-322 and Arg-323 are shown in *blue* and *pink*, respectively. *B*, stopped flow nucleotide binding experiments. Kinetic fluorescence traces upon direct mixing of NBD1-M R322A/R323A and MANT-dADP are shown (*left*). The final concentration of protein is 1 μm in all cases. The final MANT-dADP concentration ranges from 5 to 25 μm. Single exponential fits are shown as *colored lines*. Similar traces were obtained for the single mutants R322A and R323A. The rate constants *k*_obs_ obtained from fitting the kinetic traces are plotted against the MANT-dADP concentration (*right*). The association rate constants *k*_on_ for MANT-dADP binding were obtained from the slope of the linear functions. The dissociation rate constants *k*_off_ can be estimated from the *y* axis intercepts, but they were determined separately in dissociation experiments, as described under “Experimental Procedures.” Nucleotide binding parameters extracted from these data are listed in Table 1. *C*, fluorescence equilibrium titrations. NBD1-M was incubated with MANT-dADP and subsequently titrated with ADP or ATP, respectively. For the ATP titration, phosphoenolpyruvate and pyruvate kinase were present as an ATP-regenerating system. The volume-corrected data were fitted with the cubic equation for competing ligands (31), using *K_D_*(MANT-dADP) as an input value. This figure shows the titrations for NBD1-M R322A; similar curves were obtained for all mutants at different protein concentrations. Nucleotide binding parameters extracted from these data are listed in Table 1. *D*, analytical gel filtration with SLS analysis. Elution profiles of NBD1-M in nucleotide-free buffer (*red*) and with 2 mm ADP (*blue*) and 2 mm ATP (*green*) present in the running buffer are shown for NBD1-M wild type (*top*) and NBD1-M R322A/R323A (*bottom*). The ATP-containing buffer was supplemented with phosphoenolpyruvate and pyruvate kinase as an ATP-regenerating system. *Solid line*, refractive index signal; *dotted line*, calculated molecular mass of the eluted species. The actual molecular mass of the NBD1-M monomer is 45 kDa. *E*, correlation between oligomeric state and ATPase activity. The steady-state ATPase turnover (*orange circles*) and the molecular mass of oligomeric NBD1-M species (*green bars*) measured by DLS are plotted in the same diagram for different protein concentrations. The DLS experiments were performed in the presence of 2 mm ATP and phosphoenolpyruvate and pyruvate kinase as an ATP-regenerating system. The increase in ATPase activity correlates with the formation of NBD1-M trimers (*F*), which represent the smallest ATP hydrolysis-competent unit. *a.u.*, arbitrary units.

A pair of conserved, neighboring arginines in ClpB NBD1 (Arg-322 and Arg-323) is located at the interface between two subunits in the oligomeric ClpB complex (7). Both residues are in close proximity to the ATP molecule bound to the neighboring active site and were shown to be essential for the catalytic activity (17). In order to gain insights about the mechanistic role of these arginines, we decided to work with the truncated ClpB construct NBD1-M, which comprises only the N-terminal nucleotide binding domain (NBD1) and the helical M-domain of ClpB (Fig. 1*A*). We showed recently that this separately expressed construct is a fully active ATPase displaying a strong coupling between nucleotide binding and oligomerization as well as a highly cooperative ATPase activity, thereby reflecting important properties of full-length ClpB (25). We replaced both conserved arginine residues by alanine in ClpB NBD1-M. In agreement with previous experiments on full-length ClpB by Yamasaki *et al.* (17), the single and double mutants R322A, R323A, and R322A/R323A, respectively, showed about 1000-fold reduced ATPase activity compared with the wild type, indicating that both conserved arginines are crucial for ATP hydrolysis in ClpB NBD1. Next, we tested whether the mutations R322A and R323A influence the nucleotide binding behavior of ClpB NBD1-M. First, we performed stopped flow experiments to extract the kinetic nucleotide binding parameters for fluorescently labeled MANT-dADP (Fig. 1*B* and Table 1). Direct mixing and dissociation experiments showed that both the single and double mutants were able to bind MANT-dADP with similar affinities compared with the wild type. This result is in agreement with previous studies on full-length ClpB (17). Furthermore, we determined the binding affinities for the unlabeled nucleotides ADP and ATP by displacement titrations at low and high protein concentration (2 and 20 μm, respectively) (Fig. 1*C* and Table 1). Notably, the nucleotide binding affinities of the single mutants R322A and R323A and the double mutant R322A/R323A did not increase at higher protein concentrations as observed for the wild type, indicating that the conserved arginines are involved in coupling nucleotide binding and oligomerization. To further substantiate this hypothesis, we characterized the oligomerization behavior upon nucleotide binding for the single and double mutants using both DLS and SLS experiments (Fig. 1*D* and Table 1). In the presence of ATP, the wild-type protein NBD1-M oligomerizes. With increasing protein concentration, a shift toward trimeric species is observed, which correlates tightly with the increase in ATP hydrolysis rates (Fig. 1*E*). This together with Hill coefficients higher than 2.5 indicates that the trimer represents the smallest hydrolysis-competent unit (Fig. 1*F*). The nucleotide-induced oligomerization of NBD1-M is severely impaired by the R322A and R323A mutations. In contrast to the observed trimers for the wild-type protein, the molecular masses obtained for the single and double mutants indicate only a monomer/dimer equilibrium (Fig. 1*D* and Table 1). Due to substantial dilution during gel filtration, the molecular masses obtained from SLS data are not as high as in the DLS measurements performed at about 10-fold higher protein concentration. Still, the nucleotide-induced shift of the elution peak is suppressed for the mutated variants, and the obtained masses are significantly lower. It can be concluded that, although the conserved arginines Arg-322 and Arg-323 are not essential for nucleotide binding competence, they mediate the coupling between nucleotide binding and oligomerization. They are key structural elements required for the nucleotide-induced oligomerization of ClpB, a prerequisite for activity.

**TABLE 1:**
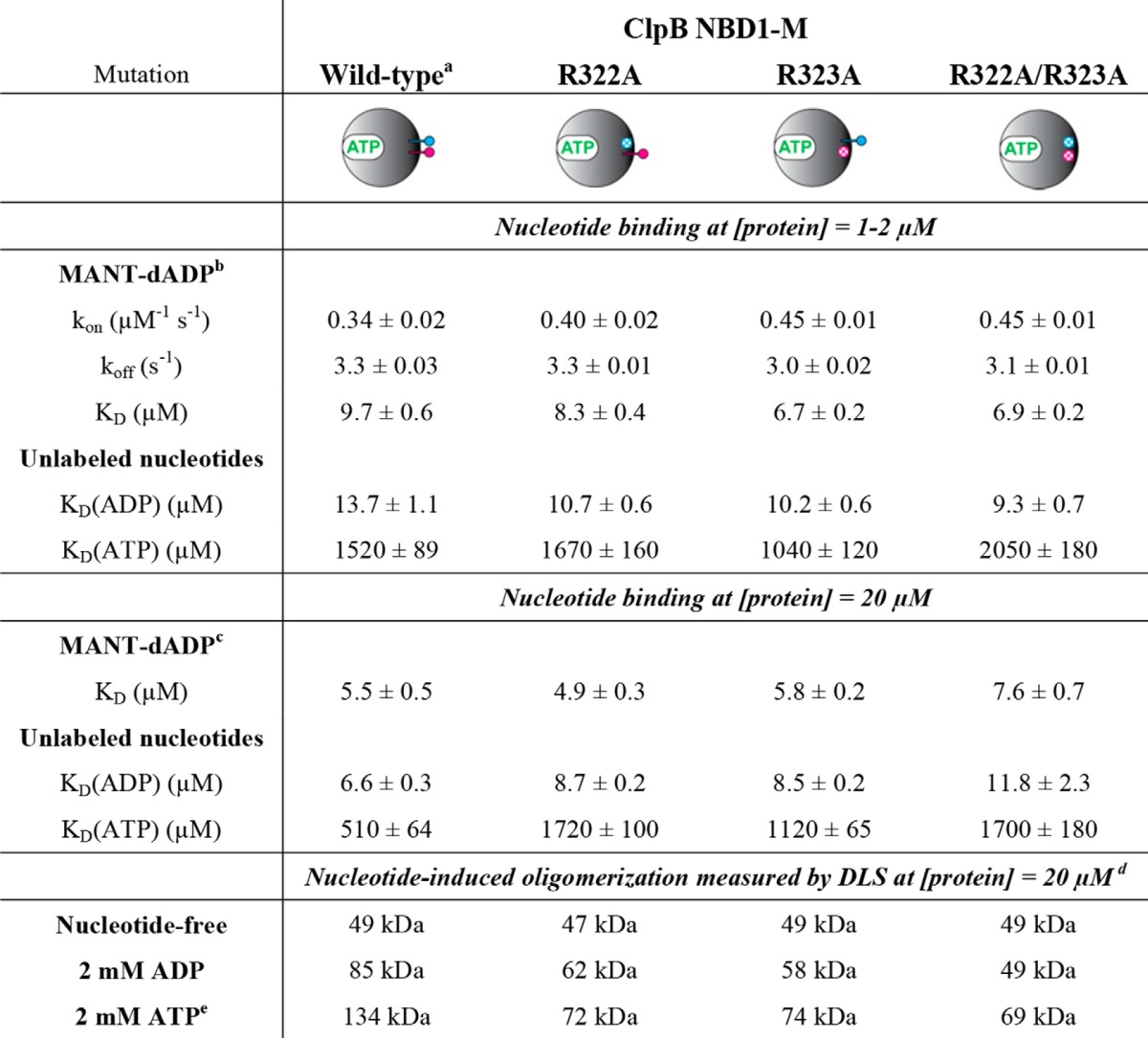
**Nucleotide binding and oligomerization parameters of ClpB NBD1-M wild type, R322A, R323A, and R322A/R323A**

*^a^* The characterization of ClpB NBD1-M wild type has been published previously (25). The parameters are given here for comparison.

*^b^* From stopped flow experiments.

*^c^* From fluorescence equilibrium titrations.

*^d^* Measurements were performed in the absence and presence of nucleotide. The molecular mass of monomeric NBD1-M is 45 kDa.

*^e^* ATP-containing solutions were supplemented with phosphoenolpyruvate and pyruvate kinase as an ATP-regenerating system.

##### Covalently Linked ClpB Dimers Facilitate Mechanistic Studies on Allosteric Regulation

**FIGURE 2.**
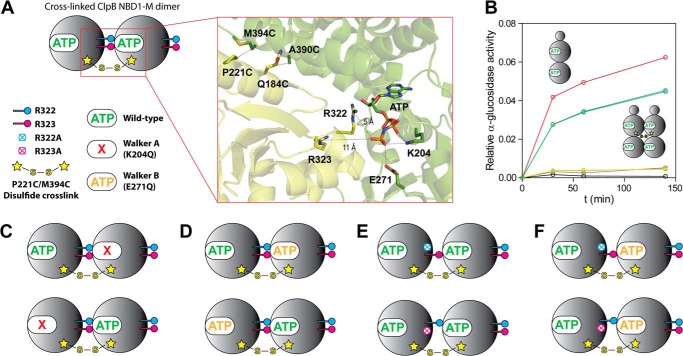
**Covalently linked ClpB NBD1-M dimer variants with intermolecular disulfide bonds.** *A*, schematic representation of a disulfide-linked NBD1-M dimer. The structural *close-up view* is based on the available crystal structure of ClpB (Protein Data Bank code 1QVR) and shows the catalytic site located in the interface between two NBD1 subunits (*green* and *yellow*, respectively) with the conserved, *trans*-acting arginines Arg-322 and Arg-323 in close proximity to the ATP molecule bound to the neighboring subunit. Lys-204 and Glu-271 are part of the catalytically essential Walker A and Walker B motifs, respectively. Two pairs of cysteines, P221C/M394C and Q184C/A390C, were introduced by site-directed mutagenesis to form intermolecular disulfide bonds, but only the first pair was used in further experiments. Different mutated dimer variants were designed, as shown in *C–F. B*, disaggregation activity of a full-length ClpB dimer cross-linked by the P221C/M394C disulfide bond. The relative α-glucosidase activity (normalized against the positive control) for different time points during the ClpB/DnaK/DnaJ/GrpE-assisted disaggregation reaction is shown for the unlinked ClpB wild-type protein (*red*) and the cross-linked ClpB dimer (*blue*). Negative controls were as follows: no chaperones present (*black*), only ClpB present (wild type (*gray*) and cross-linked dimer (*yellow*)), and only DnaK/DnaJ/GrpE present (*brown*). *C*, NBD1-M dimers with Walker A mutation K204Q either in the cross-linked interface or in the free interface. The mutation leads to a nucleotide binding-deficient catalytic site. *D*, NBD1-M dimers with Walker B mutation E271Q either in the cross-linked interface or in the free interface. The mutation leads to an ATP hydrolysis-deficient catalytic site that remains nucleotide binding-competent. *E*, NBD1-M dimers with R322A or R323A mutation in the cross-linked interface. *F*, NBD1-M dimers with R322A or R323A mutation in combination with Walker B mutation E271Q in the cross-linked interface.

With the intention to generate a well defined and fixed ClpB subunit interface, we designed covalently linked NBD1-M dimers with an intermolecular disulfide cross-link (Fig. 2*A*). Two pairs of cysteines were tested, namely P221C/M394C and Q184C/A390C. The positions were chosen on the basis of an available planar, hexameric ClpB model (30) using the program SSBOND, which suggests positions for cysteine pairs according to the optimal distances and dihedral angles for disulfide bonds (33). Whereas the Q184C/A390C dimer was severely impaired in ATPase activity (100-fold lower than the unlinked wild-type, data not shown), the P221C/M394C dimer showed ATP hydrolysis rates comparable with the wild-type. Furthermore, we tested whether the intermolecular disulfide bond between P221C and M394C affects the chaperone activity of ClpB. To this end, we generated an identically cross-linked variant of the full-length protein, which was active in an α-glucosidase disaggregation assay with about 70% activity of the unlinked wild-type protein (Fig. 2*B*). Thus, the P221C/M394C disulfide cross-link was considered “minimally invasive” and was used for all further experiments.

**FIGURE 3.**
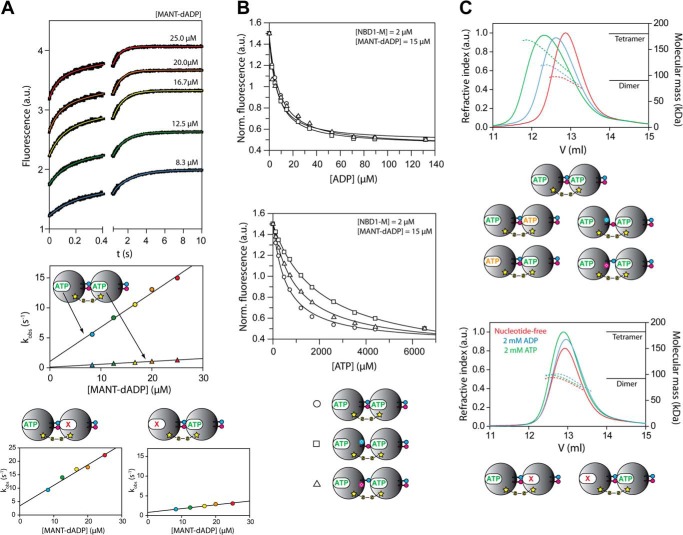
**Nucleotide binding and oligomerization of covalently linked ClpB NBD1-M dimer variants.** *A*, stopped flow nucleotide binding experiments. Direct mixing of cross-linked wild-type NBD1-M dimer and MANT-dADP leads to biphasic kinetic traces. Double exponential fits are shown as *colored lines*. The obtained rate constants *k*_obs_ plotted against the MANT-dADP concentration result in two linear functions (*circles* and *triangles*). In order to assign the respective association rate constants *k*_on_ and dissociation rate constants *k*_off_ to the two different binding sites, cross-linked NBD1-M dimers with Walker A mutations (see Fig. 2*C*) were used, which showed only monophasic kinetic traces upon direct mixing with MANT-dADP. Nucleotide binding parameters extracted from these data are listed in Table 2. *B*, fluorescence equilibrium titrations. Cross-linked NBD1-M dimer variants were incubated with MANT-dADP and subsequently titrated with ADP or ATP, respectively (wild type (*circles*), R322A (*squares*), and R323A (*triangles*)). For the ATP titrations, phosphoenolpyruvate and pyruvate kinase were present as an ATP-regenerating system. The volume-corrected data were fitted with the cubic equation for competing ligands (31), using *K_D_*(MANT-dADP) as an input value. Nucleotide binding parameters extracted from these data are listed in Table 2. *C*, analytical gel filtration with SLS analysis. Elution profiles of cross-linked NBD1-M dimers in nucleotide-free buffer (*red*) and with 2 mm ADP (*blue*) and 2 mm ATP (*green*) present in the running buffer are shown. *Top*, nucleotide-induced association of cross-linked, dimeric species was observed for wild type, Walker B mutants, and mutants of the conserved arginines (only one representative data set is shown). *Bottom*, mutants carrying a Walker A mutation do not form higher oligomers (only one representative data set is shown). The different cross-linked dimer variants are illustrated schematically, as introduced in Fig. 2. The ATP-containing buffer was supplemented with phosphoenolpyruvate and pyruvate kinase as an ATP-regenerating system. The refractive index signal is shown as a *solid line*, and the calculated molecular mass of the eluted species is shown as a dotted line. The actual molecular mass of the NBD1-M monomer is 45 kDa. *a.u.*, arbitrary units.

First, we determined the nucleotide binding parameters of the cross-linked NBD1-M dimer. Stopped flow experiments showed a biphasic fluorescence signal change upon direct mixing with MANT-dADP (Fig. 3*A*). Both kinetic phases were nucleotide concentration-dependent, indicating the presence of an asymmetric dimer with two unequal nucleotide binding sites. In order to assign the observed phases, we utilized NBD1-M dimers carrying the Walker A mutation K204Q in one of the two subunits (Fig. 2*C*). These dimers are deficient in nucleotide binding either in the cross-linked or the free active site. Indeed, MANT-dADP binding was monophasic for both variants but with significantly different kinetic parameters (*k*_on_, *k*_off_, and *K_D_*), which allowed an unambiguous assignment (Table 2). Notably, *K_D_*(MANT-dADP) is significantly lower compared with the unlinked NBD1-M wild type for both active sites of the cross-linked dimer, mainly due to a decrease in the dissociation rate constant *k*_off_. This finding again confirms the strong coupling between nucleotide binding and oligomerization in ClpB NBD1. Both association (*k*_on_) and dissociation (*k*_off_) of nucleotide are slower for the cross-linked active site than for the free one. We furthermore determined the binding affinities for unlabeled ADP and ATP by fluorescence displacement titrations and observed 10-fold stronger ADP and 18-fold stronger ATP binding compared with the unlinked NBD1-M (Fig. 3*B* and Table 2).

**TABLE 2:**
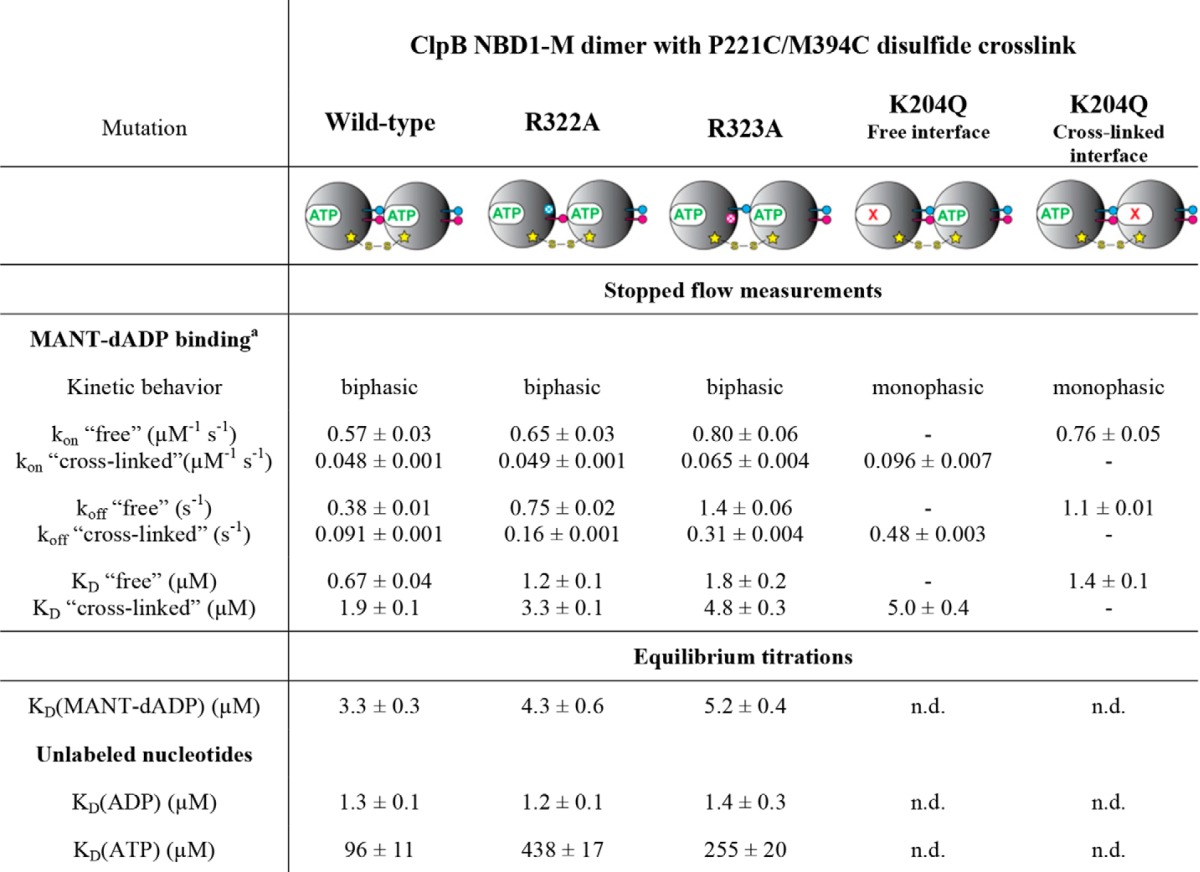
**Nucleotide binding parameters of covalently linked ClpB NBD1-M dimer variants** See Fig. 3 for corresponding experimental data. Stopped flow experiments were performed at [NBD1-M] = 1 μm in the final mixture. Equilibrium titrations were performed at [NBD1-M] = 2 μm. n.d., not determined.

*^a^* The terms “free” and “cross-linked” refer to the two different nucleotide binding sites, one being located in the free (outside) interface and the other one in the cross-linked (inside) interface.

**FIGURE 4.**
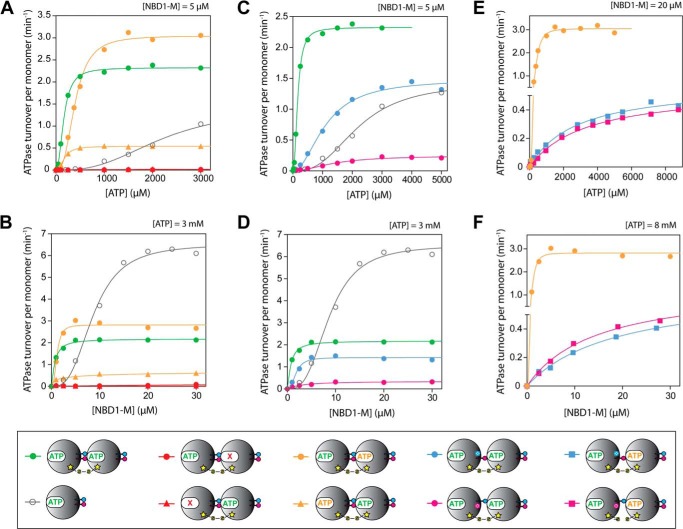
**ATPase activity of covalently linked ClpB NBD1-M dimer variants.** Steady-state ATPase turnover rates per monomer were measured at different ATP concentrations (*A*, *C*, and *E*) and protein concentrations (*B*, *D*, and *F*). The corresponding kinetic parameters *K_m_*, *k*_cat_, and Hill coefficient *n* are listed in Table 3. The *key* at the *bottom* uses the schematic representations of the different cross-linked dimer variants as introduced in Fig. 2 as follows: *gray circles*, unlinked NBD1-M wild type; *green circles*, cross-linked NBD1-M dimer wild-type; *red circles*, cross-linked NBD1-M dimer with Walker A mutation K204Q in the cross-linked interface; *red triangles*, cross-linked NBD1-M dimer with Walker A mutation K204Q in the free interface; *orange circles*, cross-linked NBD1-M dimer with Walker B mutation E271Q in the cross-linked interface; *orange triangles*, cross-linked NBD1-M dimer with Walker B mutation E271Q in the free interface; *blue circles*, cross-linked NBD1-M dimer with R322A mutation in the cross-linked interface; *pink circles*, cross-linked NBD1-M dimer with R323A mutation in the cross-linked interface; *blue squares*, cross-linked NBD1-M dimer with R322A mutation and Walker B mutation E271Q in the cross-linked interface; *pink squares*, cross-linked NBD1-M dimer with R323A mutation and Walker B mutation E271Q in the cross-linked interface.

Next, we characterized the steady-state ATPase activity of the cross-linked NBD-M dimer (Fig. 4 (*A* and *B*) and Table 3). In agreement with the observed improvement in ATP binding, the dimer showed a significantly lower *K_m_* and less pronounced dependence of the activity on protein concentration compared with the unlinked NBD1-M. However, the maximum *k*_cat_ observed for high protein concentrations of unlinked wild-type protein was not reached by the cross-linked dimer. It can be speculated that this is due to the fact that ADP release becomes rate-limiting, considering the measured *k*_off_(MANT-dADP) is lower than 0.1 s^−1^ for the cross-linked active site. We next generated NBD1-M dimers carrying the Walker B mutation E271Q in one of the two subunits (Fig. 2*D*). Using these dimers, which are fully nucleotide binding-competent but deficient in ATP hydrolysis either in the cross-linked or the free active site, we showed that indeed both of the two different active sites contribute to the overall activity of the dimer (Fig. 4 (*A* and *B*) and Table 3). When having the Walker B mutation in the free active site, the remaining activity originating from the cross-linked site is 25% of the cross-linked wild-type, whereas when mutating the cross-linked active site, the free active site is even 30% more active than the wild-type dimer carrying two intact subunits. This somewhat unexpected result emphasizes the importance of allosteric regulation. It seems that a tightly bound ATP molecule in the cross-linked active site activates the neighboring, free active site. To further study this phenomenon, we next measured the ATPase activity of the NBD1-M dimers carrying the Walker A mutation K204Q in one of the two subunits (Fig. 2*C*) that were already used to assign the nucleotide binding phases. Notably, both variants were inactive (Fig. 4, *A* and *B*). Independent of whether the cross-linked or free active site was mutated, it was sufficient to provide a nucleotide binding-deficient nearest neighbor to totally abolish the activity of the intact active site.

**TABLE 3:**
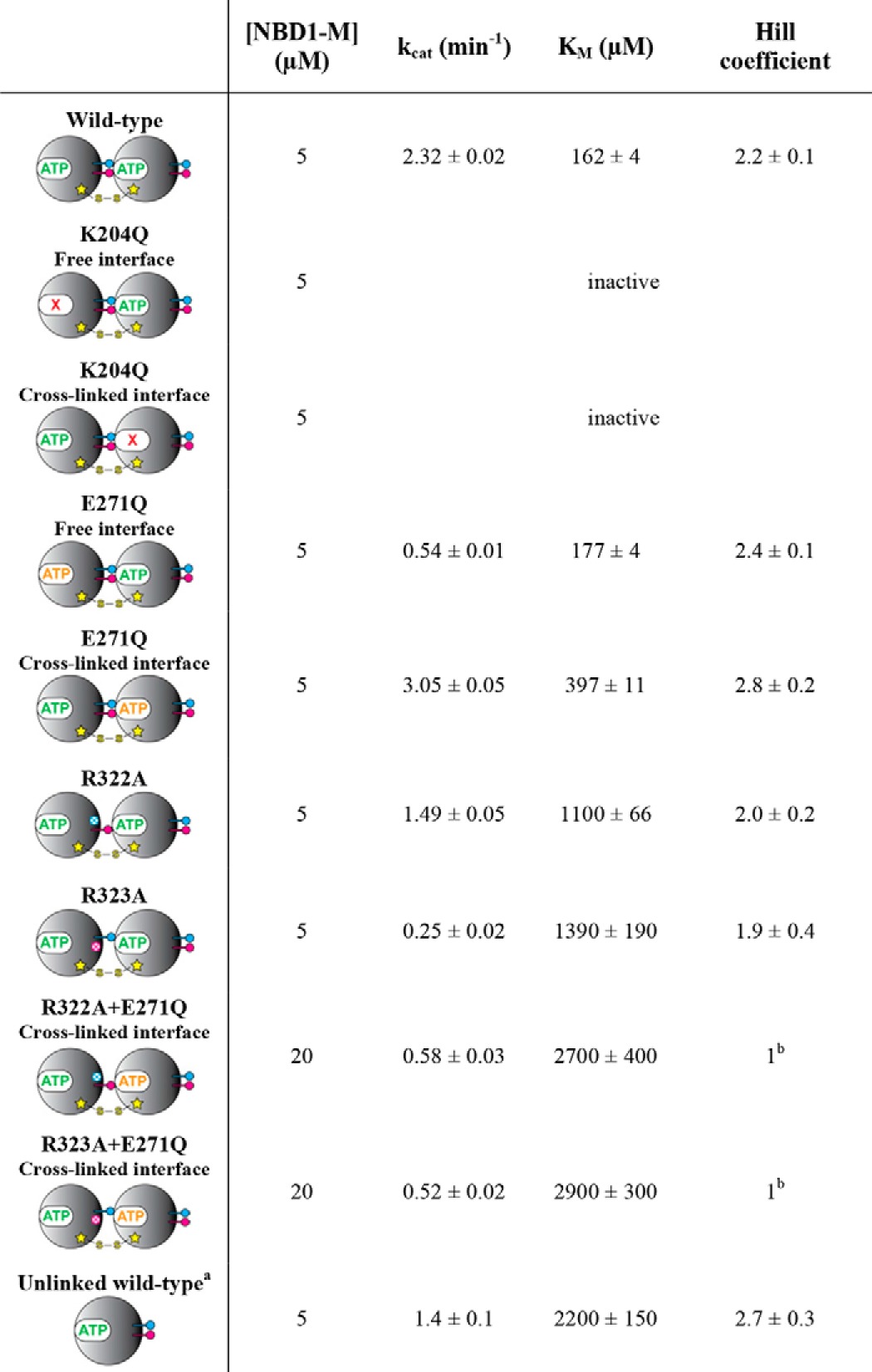
**ATPase activity parameters of covalently linked ClpB NBD1-M dimer variants** See Fig. 4, *A, C,* and *E,* for corresponding experimental data. The ATPase activity was calculated per monomer. The Hill equation was used for data fitting (Equation 1).

*^a^* The characterization of ClpB NBD1-M wild-type has been published previously (25). The parameters are given here for comparison.

*^b^* The data showed no sigmoidal shape and were therefore fitted using the Michaelis-Menten equation, which is represented by a simple hyperbolar function (Hill coefficient *n* = 1).

Furthermore, it was important to characterize the nucleotide-induced oligomerization of the cross-linked NBD1-M dimer variants. We performed analytical gel filtration runs with SLS analysis (Fig. 3*C*). The cross-linked wild-type dimer as well as both variants with the Walker B mutation showed pronounced ATP-induced oligomerization. A clear shift toward the size of tetramers was observed, indicating that also the cross-linked dimers associate with each other to form higher oligomers in the presence of ATP. In contrast, for both dimer variants carrying the Walker A mutation no nucleotide-induced oligomerization was observed, which is especially interesting for the variant with the cross-linked site being mutated. Here, the absence of nucleotide must be somehow communicated such that the intact and nucleotide binding-competent neighboring subunit cannot associate with another molecule. In summary, the cross-linked NBD1-M dimers allowed a dissection of allosteric effects in ClpB by site-specifically introducing Walker A and B mutations.

##### Allosteric Regulation between ClpB Subunits Is Communicated by Conserved Arginines

As a next step, it would be desirable to understand the molecular basis of how the observed allosteric regulation is communicated throughout the ClpB oligomer and whether the conserved arginines, Arg-322 and Arg-323, located in the subunit interfaced are involved in this task. To this end, we studied covalently linked NBD1-M dimer variants carrying either the R322A or R323A mutation in the cross-linked interface (Fig. 2*E*). This approach was chosen to distinguish a truly regulatory function of the arginines from effects associated with oligomeric stability, the latter of which were assumed to be blanked by covalently fixing the subunit interface. Indeed, SLS measurements confirmed that nucleotide-induced oligomerization of the cross-linked dimers was not impaired by the arginine to alanine mutations (Fig. 3*C*). In line with this result, both cross-linked dimer variants (R322A and R323A) were nucleotide binding-competent. The biphasic kinetic traces observed upon mixing with the fluorescently labeled MANT-dADP indicated the presence of two intact nucleotide binding sites per dimer. However, slightly higher *K_D_* values for MANT-dADP were obtained for the mutants compared with the cross-linked wild-type dimer (Fig. 3*A* and Table 2). In fluorescence displacement titrations, ADP binding was essentially not affected by the arginine to alanine mutations in the cross-linked interface, whereas ATP binding was significantly impaired (Fig. 3*B* and Table 2). This result suggests that Arg-322 and Arg-323 interact primarily with the γ-phosphate group of the ATP molecule bound to the neighboring subunit, presumably sensing the nucleotide binding state that way.

Next, we measured the steady-state ATPase activity of the NBD1-M dimers carrying the R322A or R323A mutation in the cross-linked interface (Fig. 4 (*C* and *D*) and Table 3). For both variants, the obtained *K_m_* values were significantly increased compared with the cross-linked wild-type dimer, indicating again that the conserved arginines contribute essential interactions that generate cooperativity. Notably, the R323A mutation caused a more severe loss in activity than the R322A mutation (90 and 35% decreased *k*_cat_ compared with the wild-type dimer, respectively), which might indicate that Arg-322 is mainly involved in stabilizing the subunit interface, which was provided here by the disulfide cross-link. We next asked whether the conserved arginines indeed regulate the activity of a ClpB oligomer by allosteric communication, thus affecting a neighboring catalytic site that they do not directly interact with. To this end, we combined the R322A and R323A mutation with the Walker B mutation E271Q in the cross-linked interface, which allowed studying the direct effect of the arginine mutation on the activity of the catalytic site located in the neighboring, free interface (Fig. 2*F*). Although fully competent in ATP-induced oligomerization, these dimers showed a severely impaired steady-state ATPase activity compared with the dimer carrying only the Walker B mutation (Fig. 4 (*E* and *F*) and Table 3). The cooperativity seemed to be totally blocked (Hill coefficient *n* = 1), and very high *K_m_*, low *k*_cat_, and a strong dependence on protein concentration were observed. These results clearly confirm that the conserved arginines Arg-322 and Arg-323 regulate the highly cooperative ATPase activity of ClpB by allosterically communicating between neighboring subunits, which exceeds the simple mediation of ATP-induced oligomerization.

## DISCUSSION

**FIGURE 5.**
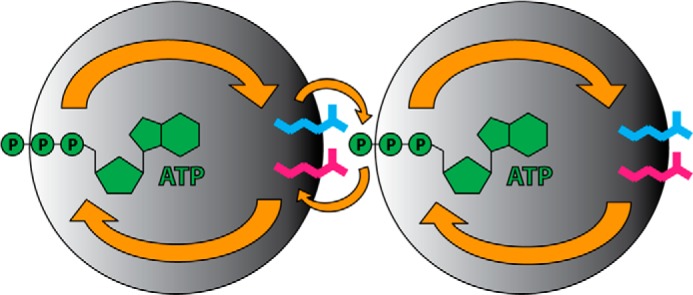
**Allosteric regulation and intersubunit communication in ClpB NBD1.** Putative pathways of allosteric regulation (*orange arrows*) are illustrated schematically. The N-terminal ATPase subunit of ClpB, NBD1, is shown as a *gray sphere*. ATP is depicted in *green* and the *trans*-acting arginines Arg-322 and Arg-323 are shown in *blue* and *pink*, respectively. The active site is located at the interface between two subunits in the oligomeric ClpB complex. The conserved arginines interact with the γ-phosphate of the ATP molecule bound to the neighboring subunit, which presumably is the structural basis for intersubunit communication. The nucleotide state of the adjacent active site is sensed and communicated throughout the AAA+ domain, which allosterically regulates the activity of the whole oligomeric complex. This process may involve other highly conserved residues that are part of nucleotide sensor motifs in *cis* and induce conformational changes throughout the ATPase cycle.

In this study, we investigated the role of the conserved, *trans*-acting arginines Arg-322 and Arg-323 in allosteric regulation and intersubunit communication in the molecular disaggregation machine ClpB (Fig. 5). Using a simplified system, namely the separate N-terminal ATPase subunit NBD1-M, it was possible to study the interplay between nucleotide binding, oligomerization, and activity in a quantitative manner. We utilized a set of well defined NBD1-M dimers with intermolecular disulfide cross-links and site-specifically introduced Walker A/B mutations to draw conclusions about allosteric effects mediated by the conserved arginine pair.

First, we showed that both arginines are involved in coupling nucleotide binding to oligomerization of ClpB NBD1-M. We identified the NBD1-M trimer as the smallest ATP hydrolysis-competent unit, which is formed upon ATP binding, but only if both arginines are present. The finding that trimer formation is essential is in agreement with previously performed mixing experiments showing that the random incorporation of two mutant ClpB subunits into the hexamer is sufficient to abolish activity (34). A previous study using full-length ClpB came to the conclusion that the *trans*-acting arginines are not involved in nucleotide binding (17). However, using the cross-linked dimers, we showed that the arginines are indeed crucial for strong and cooperative ATP binding.

The next goal was to obtain a better understanding of allosteric regulation mechanisms implemented in ClpB NBD1-M. A comprehensive mechanistic interpretation of the nucleotide binding, oligomerization, and activity data that we obtained for the different cross-linked dimer variants would greatly benefit from additional structural information about the ClpB subunit interface. The available crystal structure of ClpB from *T. thermophilus* (Protein Data Bank code 1QVR) exhibits a helical arrangement of subunits, thereby displaying a shifted subunit interface (7). The two conserved arginines Arg-322 and Arg-323 are located 5 and 11 Å away from the γ-phosphate of ATP bound to the neighboring subunit, respectively (see Fig. 2*A*), which may not reflect the active conformation. Several cryo-EM studies on ClpB and its yeast homolog Hsp104 generated models of a planar hexameric ring, which is believed to be the active form (7, 35–37). However, structural details, such as the conformation of the conserved arginines, could not be resolved. When using the hexameric crystal structure of the highly homologous AAA+ protein ClpC together with its adaptor protein MecA (Protein Data Bank code 3PXG) as a template for a planar ClpB model, both conserved arginines are at a 4–6-Å distance from a modeled ATP molecule (38). Still, at this point, there is no reliable knowledge about the exact positioning of the conserved arginine pair in the ClpB subunit interface. Thus, we put great emphasis on control experiments using different Walker A/B mutants to verify our results.

Allosteric effects related to Walker A/B mutations were studied previously, mainly by using mixing experiments (34, 39–41). The extensive amount of experimental data was complemented recently by a computational study simulating cooperativity in AAA+ proteins (42). Franzmann *et al.* (39) analyzed the allosteric network in Hsp104 and found regulatory circuits in both *cis* and *trans*. They concluded that the ATPase activity of a given NBD1 depends on the nucleotide state of the neighboring subunit, which our experiments fully agree with. However, in contrast to this previous study, we observed that the presence of a nucleotide binding-deficient subunit (Walker A mutation) inhibits ATP hydrolysis in the neighboring, intact NBD1-M unit, whereas the presence of a tightly bound ATP in a hydrolysis-deficient subunit (Walker B mutation) activates the ATPase activity of the direct neighbor. One could speculate that this regulatory feature ensures a concerted action of several subunits in the oligomeric ClpB complex, which is in agreement with previous work by DeSantis *et al.* (40) showing that ClpB indeed hydrolyzes ATP in a cooperative fashion, whereas ATP hydrolysis in Hsp104 is generally probabilistic unless the substrate is a stable amyloid.

The role of conserved, *trans*-acting arginines in AAA+ proteins was analyzed previously (16–19). Studies on ClpB and Hsp104 agree that the arginines in NBD1 are crucial for both oligomerization and activity. Notably, using our set of cross-linked ClpB NBD1-M dimers, we could (i) distinguish between regulatory functions and oligomerization effects, (ii) observe the influence of arginine mutations in a well defined environment of site-specifically engineered neighboring subunits, and (iii) be independent from overlaying allosteric effects caused by NBD2. In summary, our data indicate that the conserved arginines not only mediate the coupling between nucleotide binding and oligomerization but indeed regulate ATP hydrolysis in a truly allosteric fashion, namely by influencing a catalytic site that they do not directly interact with. Without these arginines, the cooperativity of ATP binding and hydrolysis is completely lost, even if oligomerization is ensured by chemical linkage.

It remains an open question why two such arginines are found in NBD1 of several AAA+ proteins, such as ClpB/Hsp104, ClpA, ClpC, and p97/VCP/Cdc48. Wang *et al.* (19) studied the function of this conserved arginine pair in the N-terminal AAA+ domain of p97, which is involved in various cellular processes that are directly or indirectly regulated by the ubiquitin-proteasome system. They also concluded that one arginine is more important for maintaining the hexameric state than the other, but both arginines are essential for intersubunit communication and stimulation of the ATPase activity. Clearly, both arginines are essential and cannot replace each other's function. They may have to work in concert to sense and communicate the nucleotide state and thus facilitate fine tuning of the activity in AAA+ protein complexes.

## Footnotes

2 The abbreviations used are:

NBD1: nucleotide binding domain 1
NBD2: nucleotide binding domain 2
DLS: dynamic light scattering
MANT-dADP: 2′-deoxy-3′-*O*-(*N*′-methylanthraniloyl)adenosine-5′-*O*-diphosphate
NBD1-M: ClpB construct comprising residues 141–534
SLS: static light scattering.
